# Identification and immunogenic potential of glycosylphosphatidylinositol-anchored proteins in *Paracoccidioides brasiliensis*

**DOI:** 10.3389/ffunb.2023.1243475

**Published:** 2023-08-29

**Authors:** Relber Aguiar Gonçales, Igor Godinho Portis, Thaila Fernanda dos Reis, Luiz Roberto Basso Júnior, Roberto Martinez, Heng Zhu, Maristela Pereira, Célia Maria de Almeida Soares, Paulo Sergio Rodrigues Coelho

**Affiliations:** ^1^ Life and Health Sciences Research Institute (ICVS), School of Medicine, University of Minho, Braga, Portugal; ^2^ ICVS/3B’s – PT Government Associate Laboratory, Braga, Portugal; ^3^ Laboratorio de Biologia Molecular, Instituto de Ciências Biológicas, Universidade Federal de Goiás, Goiânia, GO, Brazil; ^4^ Faculdade de Ciências Farmacêuticas de Ribeirão Preto, Universidade de São Paulo, Ribeirão Preto, Brazil; ^5^ Departamento de Biologia Celular e Molecular e Bioagentes Patogênicos, Faculdade de Medicina de Ribeirão Preto, Ribeirão Preto Medical School, Universidade de São Paulo (FMRP/USP), Ribeirão Preto, SP, Brazil; ^6^ Departamento de Clínica Médica, Faculdade de Medicina de Ribeirão Preto (FMRP), Universidade de São Paulo (USP), Ribeirão Preto, São Paulo, Brazil; ^7^ Department of Pharmacology and Molecular Sciences, Johns Hopkins University School of Medicine, Baltimore, MD, United States

**Keywords:** cell wall, GPI-anchored proteins, *Paracoccidioides brasiliensis*, PCM, Western blot

## Abstract

In fungal pathogens the cell wall plays an important role in host-pathogen interactions because its molecular components (e.g., polysaccharides and proteins) may trigger immune responses during infection. GPI-anchored proteins represent the main protein class in the fungal cell wall where they can perform several functions, such as cell wall remodeling and adhesion to host tissues. Genomic analysis has identified the complement of GPI-anchored proteins in many fungal pathogens, but the function has remained unknown for most of them. Here, we conducted an RNA expression analysis of GPI-anchored proteins of *Paracoccidioides brasiliensis* which causes paracoccidioidomycosis (PCM), an important human systemic mycosis endemic in Latin America. The expression of the GPI-anchored proteins was analyzed by quantitative PCR in both the mycelium and yeast forms. qPCR analysis revealed that the transcript levels of 22 of them were increased in hyphae and 10 in yeasts, respectively, while 14 did not show any significant difference in either form. Furthermore, we cloned 46 open reading frames and purified their corresponding GPI-anchored proteins in the budding yeast. Immunoblot and ELISA analysis of four purified GPI-anchored proteins revealed immune reactivity of these proteins against sera obtained from PCM patients. The information obtained in this study provides valuable information about the expression of many GPI-anchored proteins of unknown function. In addition, based on our immune analysis, some GPI-anchored proteins are expressed during infection and therefore, they might serve as good candidates for the development of new diagnostic methods.

## Introduction

Fungal cell walls are dynamic structures in the cell outer layer and play crucial roles in cell viability, shape, and morphogenesis (Gow et al., 2017). In fungal pathogens, the components of the fungal cell walls are recognized by the host immune system, triggering important innate and adaptive immune responses. The main cell wall-associated proteins are predicted to be modified by the addition of a GPI anchor (De Groot et al., 2003). The GPI-anchored proteins perform diverse and sometimes species-specific functions. This includes a wide range of activities varying from adhesion to host cells and tissues to cell wall biosynthesis, protection against environmental stresses, and epitope masking to avoid immune recognition (Gow et al., 2017). Genome analysis has revealed the complement of GPI-anchored proteins for many fungal pathogens. Many of these proteins appear to be rapidly evolving and species-specific; however, for most of them their role in pathogenicity remains unknown (Coronado et al., 2007).

PCM is the eighth leading cause of death among infectious and parasitic diseases and has the highest mortality rate among systemic mycoses (Colombo et al., 2011). *Paracoccidioides* spp. causes paracoccidioidomycosis (PCM), a disease affecting at least 10 million people in subtropical regions of Latin America (Brummer et al., 1993; San-Blas et al., 2002). Brazil has the highest number of endemic areas (South, Southeast and Midwest regions), which are located in the South, Southeast, and Midwest regions, and it is responsible for 85% of all cases (Restrepo, 1985; Verli et al., 2005; Martinez, 2017). *Paracoccidioides* species are the etiological agents of human paracoccidioidomycosis (Hahn et al., 2022). *Paracoccidioides* genus includes *P. brasiliensis*, *Paracoccidioides lutzii*, *Paracoccidioides americana*, *Paracoccidioides restrepiensis*, and *Paracoccidioides venezuelensis*. The disease ranges from skin lesions to invasive infections (Teixeira et al., 2009; Turissini et al., 2017).

These are dimorphic fungi that grow as filaments at mild temperatures up to 25-28°C and as pathogenic yeast cells at 35-37°C (Bocca et al., 2013). In the environment, conidia released from mycelia are inhaled by the host where they germinate in the alveolus into multibudding yeast cells.

The composition of the cell wall in *Paracoccidioides* spp. varies between morphological states, and this transition process may be essential for pathogenicity (Puccia et al., 2011; Longo et al., 2013). For instance, β-linked glucans are found only in the mycelial cell wall (San-Blas and San-Blas, 1977), while in pathogenic yeasts, 95% of the glucans are α1,3-linked, and only 5% are β-1,3-glucans (Puccia et al., 2011). The α-glucan in yeast cell walls is believed to mask epitopes from host immune recognition (Puccia et al., 2011).

It is also possible that many proteins, in particular those cell wall proteins that are differentially expressed in the pathogenic yeast cell or during the morphogenesis, can play important roles in cell wall morphogenesis and pathogenesis (Puccia et al., 2017). Our group has characterized a GPI-anchored protein (PbPga1) that is up-regulated in the pathogenic yeast phase and specifically recognized by PCM (Valim et al., 2012). This study suggests that PbPga1 may play a role in *P. brasiliensis* cell wall morphogenesis and during infection in triggering innate and humoral immunity.

A comprehensive genome analysis performed by Gonçales and colleagues (2021) identified a total of 100 Open Reading Frames (ORFs) coding for predicted GPI-proteins. Among these, 57 ORFs were associated with a known function, while 43 ORFs remain functionally uncharacterized functions in *Paracoccidioides* spp. It is possible that these genes are expressed under specific growth conditions. Studies by a number of groups on transcriptomic and proteomic analysis also revealed many predicted GPI-anchored proteins preferentially expressed in the yeast phase (Nunes et al., 2005; Bastos et al., 2007).

Despite rapid progresses in fungal genome sequening coupled, in silico annotation, and the partial characterization of *Paracoccidioides* spp. GPI-anchored proteins, their role in the pathogenic process remains largely unknown, especially for those species-specific proteins. Here, we applied two different approaches to further characterizing GPI-proteins in *P. brasiliensis*: we analyzed the *in vitro* transcription profile for 46 GPI-protein genes in the two morphological states and verified the immunological reactivity during human infection for a selected four recombinant predicted GPI-proteins.

## Materials and methods

### Patients serum samples

This study involves using of serum samples obtained from patients as part of routine care. The Ethics Committee on Research at Hospital das Clínicas da FMRP-USP (protocol #13982/2005) did not require the study to undergo review or approval from an ethics committee because the study used sera from patients who had already undergone laboratory testing.

### Fungal strains and growth conditions

The virulent strain of *P. brasiliensis* was obtained from Gustavo H. Goldman at the University of São Paulo, Brazil and was used for all experiments in this study. Cultures were maintained on solid, modified BHI (Brain Heart Infusion) medium (Kasvi, São José dos Pinhais, Paraná, Brazil) at 37°C for the yeast phase. For the hyphal phase, cultures were grown in liquid BHI medium at 25°C and 37°C for yeast phase. Fungi were cultivated for 7 days in liquid BHI medium with shaking.

### RNA extraction

Total RNA was extracted from yeast and hyphae cells using Trizol (TRIzol RNA isolation reagents, Invitrogen, Thermofisher, Massachusetts, USA) following the manufacturer’s instructions. RNA was resuspended in DEPC-treated water, quantified by spectrophotometry, and stored at -80°C.

### Expression analysis by qRT-PCR

Total RNA was treated with DNase using the protocol provided by the manufacturer (Biolabs Inc., New England, USA). First-strand cDNA synthesis was carried out using the Improm-II reverse transcriptase (Promega Corporation, Madison, Wisconsin, USA) following the manufacturer’s instructions. Quantitative real-time PCR was performed using SYBR Green PCR master mix (Applied Biosystems, Foster City, CA, USA). The reaction conditions were as follows: 50°C for 2 min, 95°C for 5 min, and 40 cycles of 95°C for 15 sec and 60°C for 1 min. The primer concentration used in each reaction ranged from 2 to 10 pmol. The amount of cDNA used in the qRT-PCR reaction was determined empirically by diluting the samples from 1:5 to 1:100. The expression levels of the predicted genes were calculated using the ΔCt (cycle threshold) method (Kubista et al., 2006), and normalized to the expression of the L34 gene. The specificity of the reaction was confirmed by dissociation curve analysis (95°C for 15 min, 60°C for 20 sec, and 95°C for 15 sec). The results were presented as the means of duplicate measurements in three independent experiments. Negative controls were included in each round of reactions to ensure the absence of RNA or genomic DNA contamination.

### Cloning of GPI cDNAs in yeast expression vectors

To identify and express GPI-anchored proteins in *Saccharomyces cerevisiae*, we selected four ORFs based on their expression levels by western blot analysis, molecular weight similarities to predicted conservation levels in other fungi, and enhanced expression in yeast. We used the pEGH vector, which was modified to be compatible with the Gateway System to create the pEGH-A vector, and expressed the proteins as fusions with a tail of six histidine (6His) and GST (Glutathione-S-Transferase) at the N-terminus (Zhu et al., 2001). The pEGH-A vector contains a galactose-inducible promoter, an ampicillin resistance marker, and a URA3 auxotrophic marker. Transformants were plated on SC without URA solid medium (2% glucose) and incubated at 30°C for 48 h, followed by inoculation in 25 ml of SC without URA liquid medium (2% raffinose) and incubation in a shaker at 30°C and 170 rpm for 48 h. Upon reaching optimal optical density, ORF expression was induced by the addition of 4% galactose filtered for 18 h at 30°C. After induction, cells were centrifuged at 3,800 x *g* for 20 min, and the insoluble fraction was prepared for GPI-anchored protein extraction.

### Purification of recombinant GPI-anchored proteins

The purification was carried out as follows: cells expressing the target proteins were resuspended in 800 µL of lysis buffer per 289.3 mL of culture, along with 800 µL of 0.5 mm zirconium beads, and homogenized by vortexing for 10 cycles of 1 min with 20 sec intervals at 4°C. After lysis, the samples were washed with 800 µL of lysis buffer, and the supernatant was collected by centrifugation at 13,000 x *g* for 10 min at 4°C. The supernatant was further clarified by centrifugation, and the soluble extract containing the GST-fused target proteins was incubated overnight at 4°C with glutathione sepharose resin equilibrated with Buffer A. The flowthrough was collected, and the resin was washed with 12 mL of Wash Buffer, and the eluate was collected. The resin was incubated with 10 mL of elution buffer (Buffer A with 30 mg of reduced glutathione) for 3 h at 4°C under vigorous stirring, and the elution was collected. The samples were then concentrated by means of the Amicon system with three washes of 15 mL of 1 M Tris HCl pH 7.4 at 1800 x g for 40 min. The lysis buffer contained 100 mM Tris HCl pH 7.4, 1 mM NaCl, 1 mM EDTA pH 8.0, 0.1% β-mercaptoethanol, 0.5 mM PMSF, 0.1% Triton x-100, and protease inhibitor cocktail. The resin was first treated with the column buffer and then equilibrated with Buffer A. The column buffer contained 100 mM Tris HCl pH 7.4, 150 mM NaCl, 2 mM DTT, 1 mM EDTA, and 0.2 mM sodium azide.

### The immunoblot analysis

The immunoblot analysis was performed to assess the reactivity of the recombinant GPI-anchored proteins with sera from patients with various infections or healthy individuals. The polyacrylamide gel containing the proteins was transferred to a nitrocellulose membrane using a transfer buffer. Non-specific interactions were blocked by incubating the membrane with TBS-T containing skim milk powder. The membrane was then incubated with sera from patients and anti-GST antibody, and the reactivity of the recombinant GPI-anchored proteins was compared to that of the protein GP43 used in the diagnosis of paracoccidioidomycosis (PCM). After the incubation period, the membrane was washed with TBS-T, and human anti-IgG secondary antibody conjugated with peroxidase was added. The membrane was then revealed using a substrate for peroxidase and exposed to X-ray film. The sera used in the analysis were obtained from Prof. Dr. Roberto Martinez-HCFMRP-USP.

### Enzyme-linked immunosorbent assay

Polystyrene microplates (Nunc®, Denmark) were coated with recombinant proteins from *P. brasiliensis* (1 g/well), which were diluted in 0.2 M sodium carbonate buffer (pH 9.6) and incubated for 16 h at 4°C. The microplate wells were then washed three times with TBS wash buffer containing 0.05% Tween 20 (v/v) and blocked with TBS containing 0.05% Tween 20 and 3% gelatin (Difco, Merck, Sigma-Aldrich®, Darmstadt, Germany) (200 µL/well), and incubated for 2 h at 37°C. After blocking, the plates were incubated for 2 h at 37°C with patient sera. The wells were then washed and incubated for 1 h at 37°C with human IgG conjugated with peroxidase. The wells were washed again, and the reaction was developed with a substrate solution for peroxidase, which contained 5 mg ortho-phenylenediamine (OPD) (Merck, Sigma-Aldrich®, Darmstadt, Germany), 12.5 mL of 0.1 M citric acid (Merck), Na2HPO4 0.2 M (Synth, Sigma-Aldrich®), and 19 µL of 30% H2O2 (Synth, Sigma-Aldrich®). The reaction was stopped by adding 50 µL/well of 2 M H2SO4 (Merck, Sigma-Aldrich®, Darmstadt, Germany), and the absorbance was measured at 450 nm using an ELISA reader (PowerWave X Biotek Instruments). All trials were monitored without antigen controls, without sera of patients, and with only peroxidase conjugated to human IgG.

### Statistical analysis

The data were analyzed using the statistical program “one-way ANOVA” (analysis of variance), followed by a Tukey test to compare the means of the groups. Prism 9.3.1 software (GraphPad, CA, USA) was used for all analyses. The difference between the data was considered significant when *p* < 0.05.

## Results

The ability of dimorphic fungal pathogens, including *P. brasiliensis*, to cause disease depends on temperature-dependent morphotype transitions, and GPI-anchored proteins play a critical role in these processes. To identify the differentially expressed GPI-anchored protein genes in yeast and hyphal growth phases, we compared the RNA expression levels of 46 ORFs predicted as GPI-anchored protein coding genes in the *P. brasiliensis* genome during growth at 30°C (hyphal growth) and 37°C (yeast growth) using qRT-PCR data. We selected ORFs that exhibited at least double the expression level in yeast compared to hyphae, and vice versa. Following this selection, we performed a comprehensive heat map analysis focusing on 32 GPI-proteins that displayed significant differential expression patterns.

### Differential expression of GPI-anchored proteins in hyphal cells

**Figure 1:**
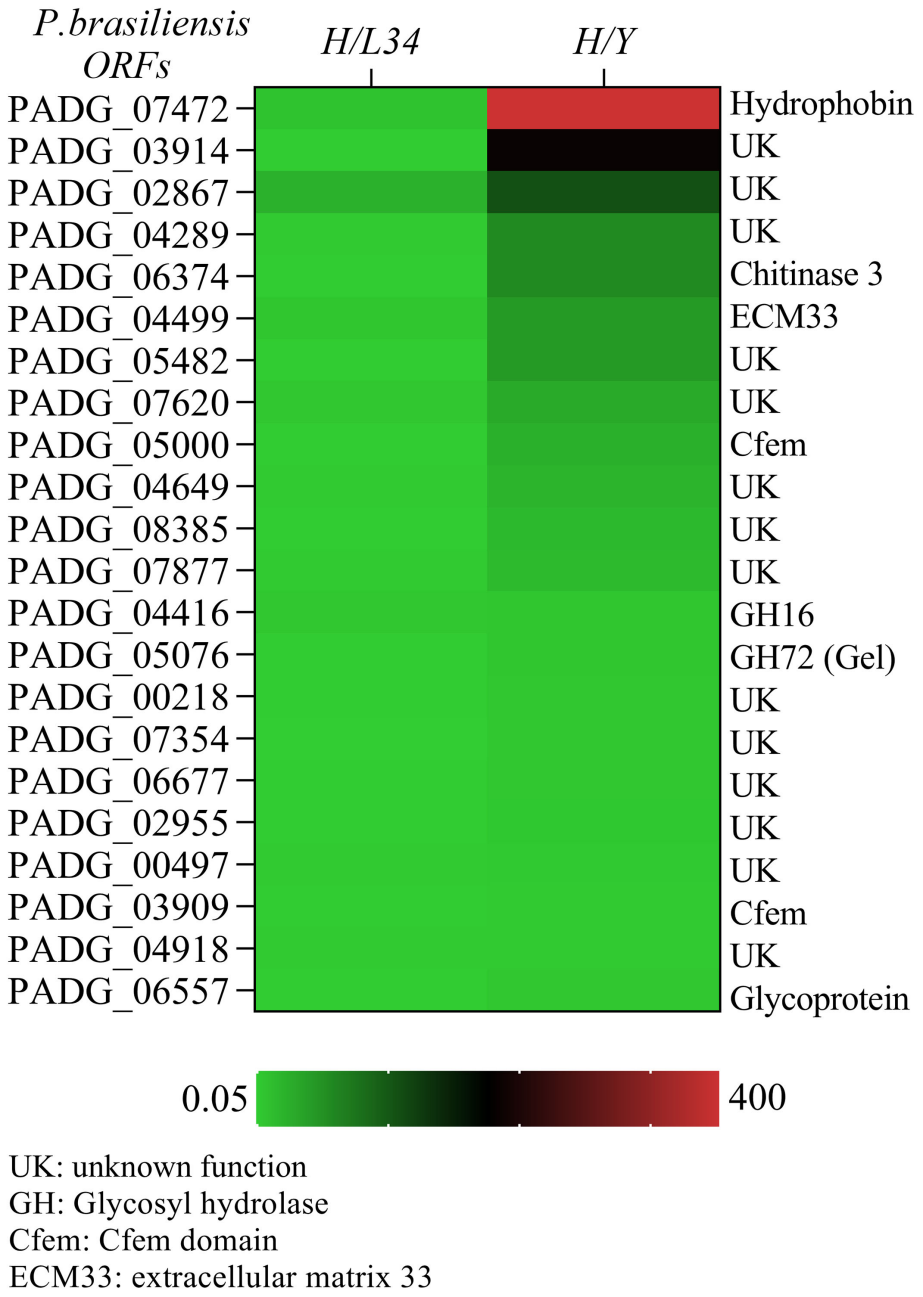
Quantitative transcriptional expression of ORFs from hyphae to yeast cell transition phase from *Paracoccidioides brasiliensis*. The color bar displays a heat map indicating the difference in abundance (ranging from 0.05 to 400-fold) of transcriptional expression intensities between yeast and hyphae in *P. brasiliensis*. The mRNA was grouped using unsupervised hierarchical clustering.

A set of 22 GPI-proteins were found to be differentially expressed (>2-fold) in hyphae as compared to yeast (H/Y) (**Figure 1**), including hydrophobin 2 (PADG_07472), which exhibited a remarkable 355-fold increase in mycelia relative to yeast and an 8.07-fold increase compared to L34 (H/L34) (**Figure 1**). Additionally, a group of GPI-proteins of unknown function, including PADG_03914, PADG_02867, PADG_04289, PADG_05482, PADG_07620, PADG_04649, PADG_08385, PADG_07354, PADG_06677, PADG_02955, PADG_06557, and PADG_00497, showed elevated expression in mycelia, indicating their potential roles in regulating the mycelial phase of *P. brasiliensis* (**Figure 1**).

Two predicted proteins with the CFEM domain, including the homolog of ECM33 (PADG_04499), a glucanosyltransferase (GEL2/PADG_05076), and a CRH-GH16 glycosylase (PADG_04416), exhibited differential expression in hyphae. Notably, the homolog of ECM33 was highly expressed in mycelium (5.48-fold increase as compared to L34) and exhibited a 44-fold increase in expression in hyphae compared to yeast (**Figure 1**). The expression of the glucanosyltransferase and CRH-GH16 glycosylase were also significantly higher in the mycelial phase (6-fold and 5.06-fold, respectively, compared to L34) and exhibited higher expression in hyphae than in yeast (**Figure 1**). We found a glycoside hydrolase (DCW1), namely PADG_1494, which showed the similar expression in mycelium and yeast cells (data not shown). These differentially expressed GPI-proteins may play important roles in the regulation of mycelial growth and morphogenesis in *P. brasiliensis*.

### Differential expression of GPI-anchored proteins in yeast cells

**Figure 2:**
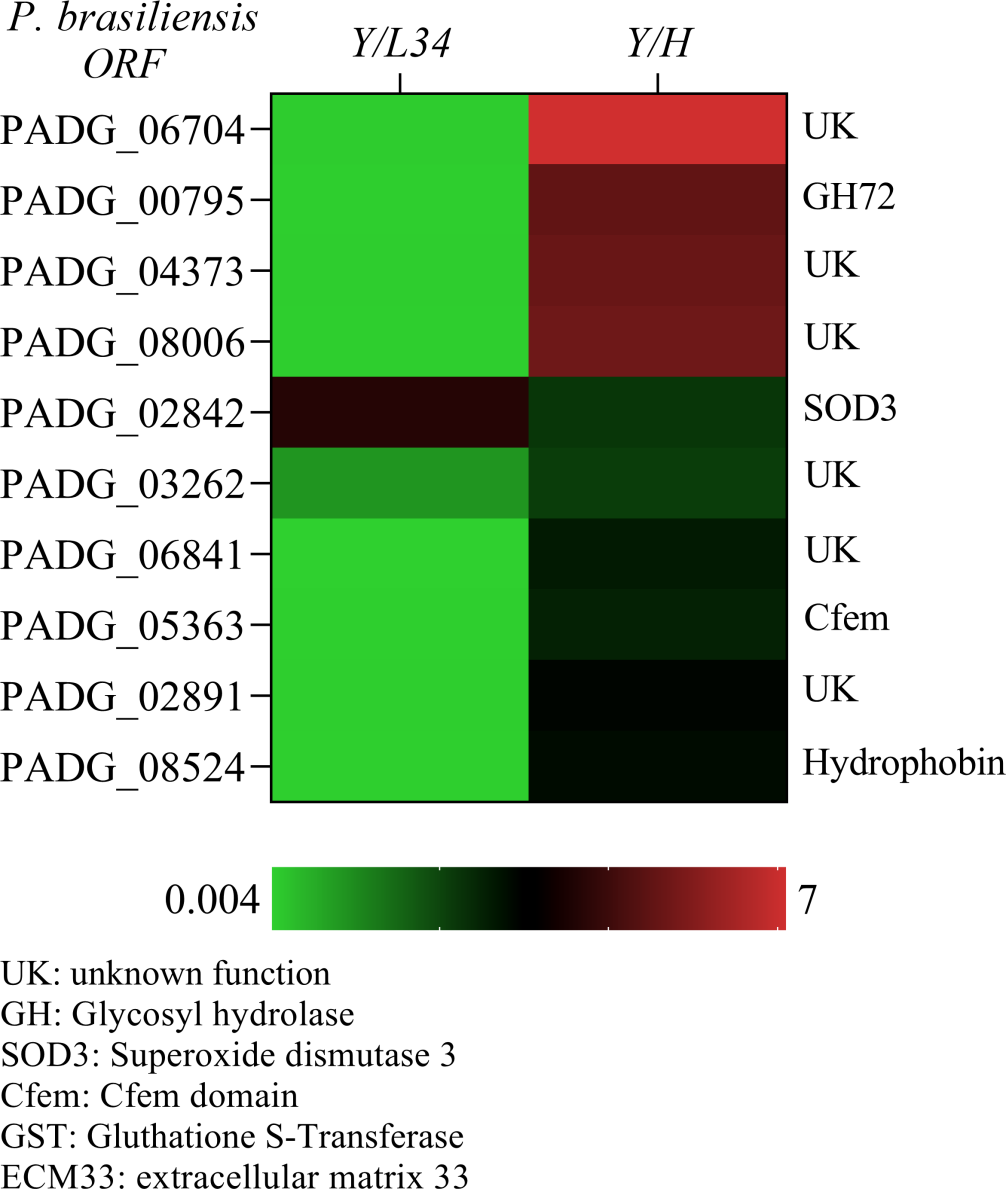
Quantitative transcriptional expression of ORFs from yeast to hyphae cell transition phase from *Paracoccidioides brasiliensis*. The color bar displays a heat map indicating the difference in abundance (ranging from 0.05 to 400-fold) of transcriptional expression intensities between yeast and hyphae in *P. brasiliensis*. The mRNA was grouped using unsupervised hierarchical clustering.

We identified a group of 10 GPI-anchored proteins that showed significantly higher expression levels in yeast cells (Y/L34), with over 2-fold induction compared to hyphal growth (Y/H) (**Figure 2**). This group includes several proteins of unknown function (UK), PADG_067040, PADG_04373, PADG_08006, PADG_03262, PADG_06841, and PADG_02891, as well as three proteins with known functions (PADG_00795, PADG_02842, and PADG_08524). Of particular interest, a member of the Gas family was found to be highly expressed in yeast cells, suggesting a potential role in the processing of glucans during this morphological phase (**Figure 2**). Further studies are needed to elucidate the specific functions of these differentially expressed GPI-anchored proteins in yeast cells.

### Reactivity of GPI-anchored proteins with serum from patients with PCM

We cloned 46 predicted GPI-anchored proteins in a yeast expression vector and purified them as N-terminal GST-tagged proteins in a high throughput 96-well format (data not shown) (Zhu et al., 2001). We selected four GPI-anchored proteins to evaluate their immunoreactivity to sera obtained from 7 patients with paracoccidioidomycosis (PCM) and to the healthy controls, using the Western blot and ELISA analyses. Two of these proteins (i.e., PADG_04617/cutinase and PADG_01494/glycosyl hydrolase) had similar RNA expression during yeast and hyphal growth, one protein (PADG_06557, unknown function) had higher expression during hyphal growth, and one protein (PADG_02842, SOD3) had higher expression during yeast growth.

**Figure 3:**
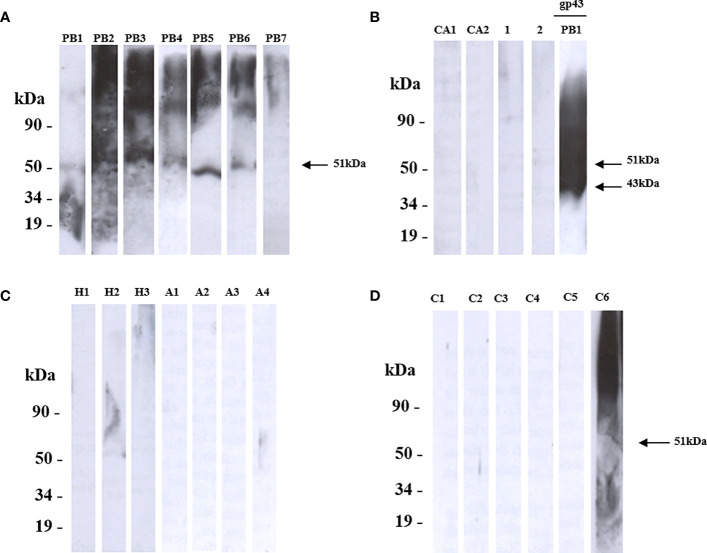
Shows the recognition of rPADG_02842 (SOD3) by sera from patients with Paracoccidioidomycosis (PCM), using SDS-PAGE and western blotting. **(A)** PB1-PB7: sera from 7 PCM patients recognized 6 polypeptides of 51 kDa (arrow), while GST-negative control and glutathione-S-transferase protein were not recognized. **(B)** Ca1 and Ca2: 2 sera from patients with candidiasis, and healthy sera from 2 persons served as negative controls (1 and 2). GP43 protein of *P. brasiliensis* native 43 kDa (14 mg/ml) was used as a positive control (arrow). **(C)** H1-H3: serum from 3 patients with histoplasmosis, and A1-A4: serum of 4 patients with aspergillosis. **(D)** C1-C6: 6 sera from unrelated diseases. All primary antibodies were diluted to 1:1000, and goat anti-human IgG and C+ GST and GST were used as secondary antibodies.

Therefore, we decided to examine the reactivity of these proteins using the Western blot analysis. We showed that rPADG_02842 was recognized by sera from six patients with PCM (PB1-PB7) and showed no reactivity to serum sample of those patients diagnosed with histoplasmosis, aspergillosis, or candidiasis (**Figures 3A–D**). Since one patient serum (C6) showed non-specific reactivity, we investigated the patient’s history and found that this patient is presented fungal pneumonia, possibly caused by *Paracoccidioides* spp. (**Figure 3D**). PADG_02842 has homology to superoxide dismutase (SOD3) in other organisms and has counterparts in 12 other fungi close to *P. brasiliensis* and *P. lutzii*, including *Blastomyces dermatitidis, Histoplasma capsulatum, Coccidioides immitis, Microsporum canis, Trichophyton rubrum, Aspergillus nidulans, Penicillium marneffei, Neurospora crassa, Fusarium oxysporum, Magnaporthe grisea, and Candida albicans* (Gonçales et al., 2021). Recent studies by Tamayo and colleagues (2016) have shown that PADG_02842 is a virulence factor in *P. brasiliensis* in knockdown studies (Tamayo et al., 2016). The present study found that PADG_02842 has 15.9% serine and threonine and 9 predicted domains for *N*-glycosylation and 5 for *O*-glycosylation. Despite the presence of homologs in other fungi and a small number of glycosylation sites, this protein showed specificity for patients with PCM.

**Figure 4:**
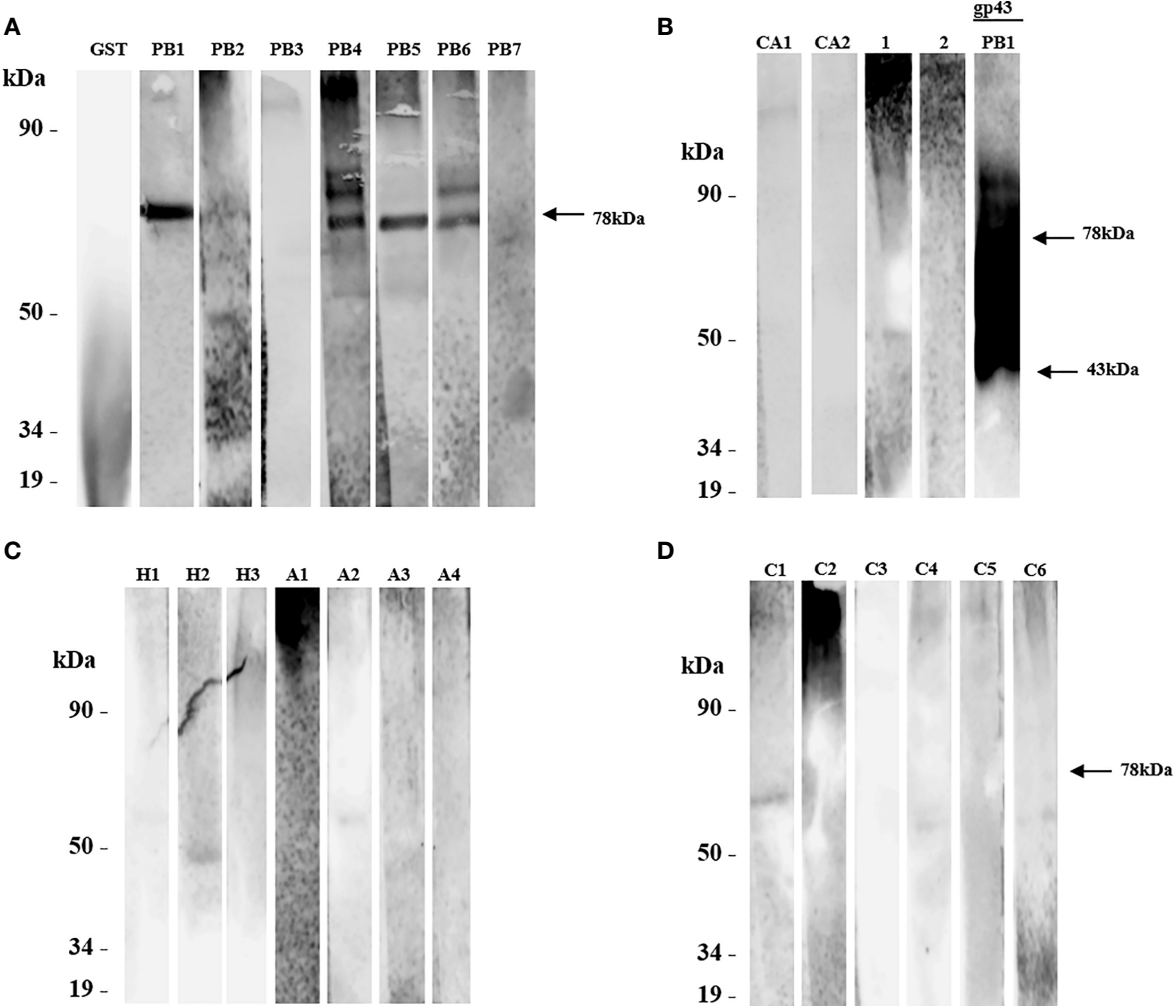
The protein rPADG_01494 was evaluated for recognition by sera from patients with paracoccidioidomycosis (PCM) using SDS-PAGE and western blot. **(A)** PB1-PB7: sera from 7 PCM patients were tested, with only a polypeptide of 78 kDa being recognized in PB1, PB2, and PB5 sera (arrow), and 2 polypeptides recognized in PB4 and PB6 sera. No reactivity was observed with sera from 2 patients PB3 and PB7. GST-negative control and glutathione-S-transferase protein were not recognized. **(B)** CA1 and CA2: 2 sera from patients with candidiasis, and healthy sera from 2 persons served as negative controls (1 and 2). GP43 protein of *P. brasiliensis* native 43 kDa (14 mg/ml) was used as a positive control (arrow). **(C)** H1-H3: serum from 3 patients with histoplasmosis, with H2 serum reacting with a polypeptide of about 50 kDa. A1-A4: serum of 4 patients with aspergillosis, with A2 serum reacting with a polypeptide of about 50 kDa, and A1 serum showing nonspecific reactivity. **(D)** C1-C6: 6 sera from unrelated diseases. All primary antibodies were diluted to 1:1000, and goat anti-human IgG and C+ GST and GST were used as secondary antibodies.

Our analysis showed that rPADG_01494 was recognized by five sera from patients with PCM (PB1, PB2, PB4, PB5, and PB6) (**Figure 4A**). In some of these samples, a fragment predicted for this protein was also detected. rPADG_01494 showed no cross-reactivity with sera from patients with histoplasmosis (H1 and H2), aspergillosis (A2), candidiasis (CA1 and CA2) (**Figures 4B, C**), or unrelated diseases (C1, C4, and C6) (**Figure 4D**). In these samples, fragments of different molecular weights to the predicted rPADG_01494 were also detected. As noted in other recombinant proteins, serum of a patient diagnosed with fungal pneumonia (C6) showed cross-reactivity (**Figure 4D**). PADG_01494 is predicted in other organisms as a glycoside hydrolase (DCW1) and is conserved in 15 other fungi, including *B. dermatitidis, H. capsulatum, C. immitis, M. canis, T. rubrum, A. nidulans, P. marneffei, U. reesi, N. crassa, F. oxysporum, M. grisea, C. albicans, S. cerevisiae, S. pombe, and C. neoformans* (Gonçales et al., 2021). The ORF shows 15.3% serine and threonine, with 5 possible domains for *N*-glycosylation and 10 for *O*-glycosylation.

The Western blot analysis showed that rPADG_06557 was recognized by sera from six patients with PCM (data not shown). In addition, rPADG_06557 was recognized by a serum of a histoplasmosis patient (H2) and a candidiasis patient (CA2) and showed no reactivity to sera of patients with aspergillosis (data not shown). However, sera from two patients with unrelated diseases (C4 and C6) showed nonspecific reactivity (data not shown). PADG_06557 is of unknown function and is preserved only in three other fungi: *A. nidulans, M. canis, and C. immitis* (Gonçales et al., 2021). The ORF shows 24.1% serine and threonine, with 6 possible domains for *N*-glycosylation and 71 for *O*-glycosylation.

The rPADG_04617 protein was recognized by six sera from patients with PCM in our Western blot analysis. The protein showed cross-reactivity with sera from patients with histoplasmosis (H2), aspergillosis (A1) or unrelated diseases (C1, C2, and C4) (data not shown). Similarly, two proteins, namely rPADG_02842 and rPADG_04617, showed non-specific reactivity against serum from a patient diagnosed with pneumonia (C6). PADG_04617 is predicted in other organisms as a possible protein belonging to the family of cutinases. This protein is conserved in 10 other fungi, including *B. dermatitidis, H. capsulatum, C. immitis, M. canis, T. rubrum, P. marneffei, U. reesi, N. crassa, F. oxysporum, and M. grisea* (Gonçales et al., 2021). The percentage of serine and threonine is 13.3%, with 3 possible domains for *N*-glycosylation and *O*-glycosylation.

### Confirming the immunoreactivity with ELISA

To firmly establish a sensitive diagnostic test for *Paracoccidioides* spp., it is important to identify the epitopes produced by the pathogen in the blood. To this end, we analyzed the reactivity of recombinant GPI-anchored proteins (rGPI-proteins) using an enzyme-linked immunoenzymatic assay. Specifically, we examined the reactivity of four rGPI-proteins, including rPADG_02842, rPADG_04617, rPADG_01494, and rPADG_06557.

**Figure 5:**
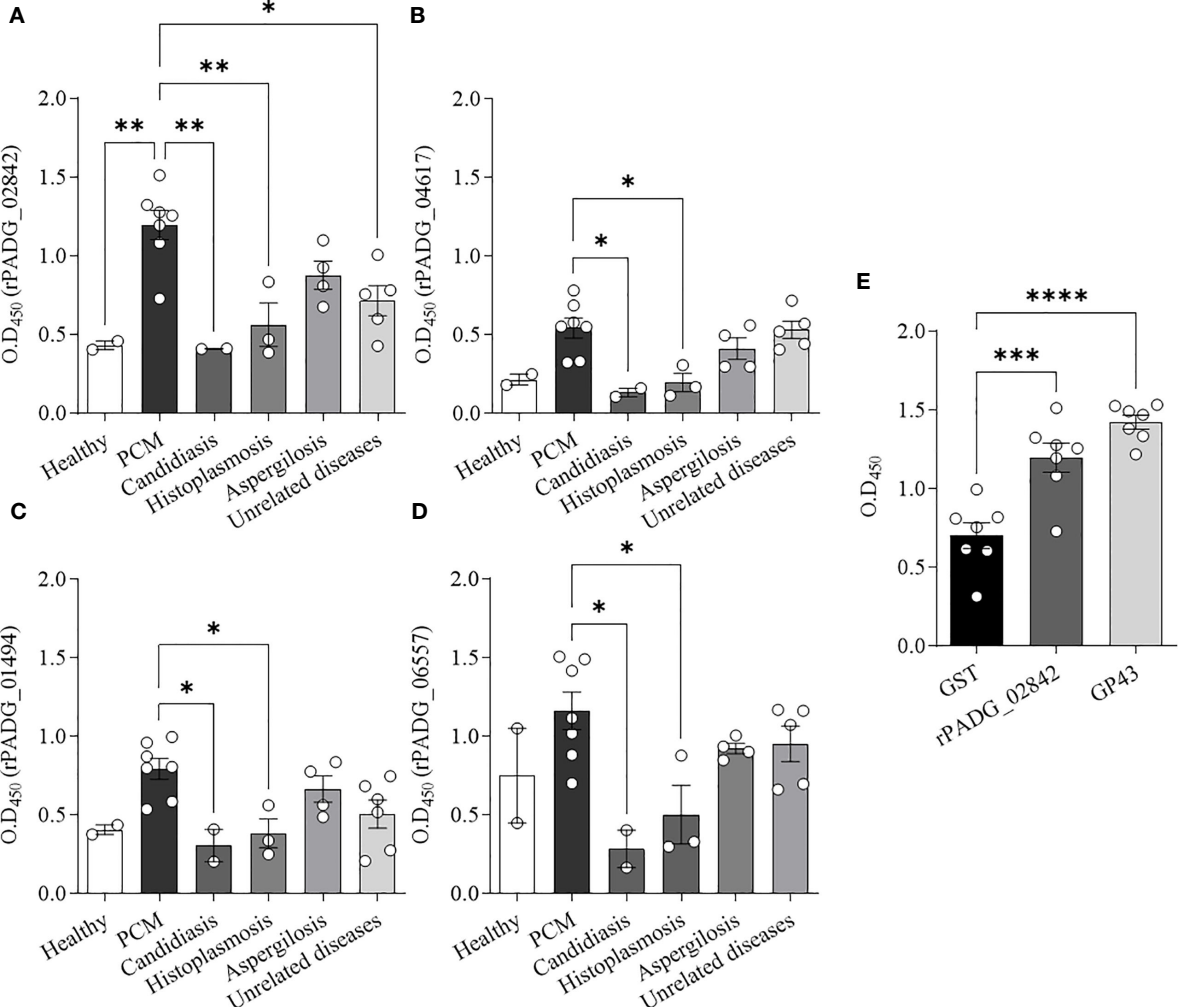
Detection of anti-rGPI-protein antibodies in sera from patients with PCM by ELISA. Recombinant GPI-proteins (1 μg/mL) were used to coat ELISA plates, and sera from different patient groups were added as primary antibodies. The results were evaluated based on optical density (OD_450_ nm) readings. **(A–D)** Seven sera from patients with PCM were tested, and the healthy control group included two sera from individuals without clinical evidence of fungal infection. Additionally, two sera from patients with candidiasis, three from patients with histoplasmosis, four from patients with aspergillosis, and sera from patients diagnosed with unrelated diseases (tuberculosis, lymphoma, icterus, pneumonia, and asthma) were tested. **(E)** Seven sera from patients with PCM were evaluated against rPADG_02842 protein, rGST, and GP43. Bars depict the mean ± SEM and the values provided by serum reactivity against recombinant proteins, and the statistical significance was determined using one-way ANOVA, where *p <0.1, **p <0.05, ***p <0.01, and ****p <0.001; bars where p is not shown, are not significant.

Our results showed that the rPADG_02842 protein (**Figure 5A**) demonstrated significant differences in reactivity compared to sera from healthy subjects, as well as sera from patients with other fungal diseases, such as candidiasis and histoplasmosis, and unrelated diseases. The other three proteins tested in this study, including rPADG_01494, rPADG_04617 and rPADG_06557 (**Figures 5B–D**), were found to exhibit significant differences in reactivity for sera from patients with PCM compared to those with candidiasis and histoplasmosis. In **Figure 5E**, the reactivity of GST is compared to the reactivity of rPADG_02842 and gp43. The rPADG_02842 and GP43 proteins exhibited statistically significant differences in reactivity compared to the GST-tag (**Figure 5E**). These proteins did not show any difference in reactivity in healthy patients’ sera, or sera from patients with aspergillosis or unrelated diseases.

## Discussion

During infection, dimorphic fungal pathogens can have morphological changes that depend on environmental factors such as host temperature and activation of specific groups of proteins. GPI-proteins are a group of proteins that are regulated at the transcription level during yeast to hypha morphogenesis and during responses to environmental challenges and possibly during fungal infections.

In this study, we identified hydrophobins as differentially expressed in hyphae hydrophobins that are important for many morphogenetic processes in fungi, including sporulation, the development of fruiting bodies, and infecting structures (**Figures 1**, **2**). In various fungi, hydrophobins such as RodA are another group of proteins are necessary to hide dormant conidia and avoid recognition by cells of the innate immune system. The hydrophobins found in *P. brasiliensis* could play an essential role in the interaction between the immune system and newly inhaled spores. We also identified proteins with cysteine domain related to the cellular surface, such as those containing CFEM domains. These proteins have been described in several species of fungi and are believed to work as surface receptors or signal transducers, or even as adherence molecules in the fungi-host interaction. PADG_03914, which contains a strong cysteine motif, demonstrated its highest differential expression in mycelium compared to L340 (**Figure 1**). The CRoW motifs are found in *C. albicans* and *C. immitis* surface antigens and are induced during the change for filamentous growth.

Research on *A. fumigatus* and *P. brasiliensis* has identified key proteins that play a role in the growth, development, and structure of fungal cells. In *A. fumigatus*, disturbances in the Gel2 protein complex resulted in slower growth, decreased β-1,3-glucan content, increased chitin, and abnormal conidiogenesis (Mouyna et al., 2005). Similarly, studies on P. brasiliensis have identified the PbGEL3 protein, which is located on the surface of fungal cells and serves as a functional homologous of Gel2p (Castro Nda et al., 2009).

Another important protein described in this work is the CRH-GH16 glycosylase (**Figure 1**), which is a predicted GPI-protein that may play a critical role in mycelium formation. Transglycosylases like Crh1p, Crh2p/Utr2p in *S. cerevisiae*, and CRH-GH16 are essential for connecting chitin to β-(1-3)-glucan and β-(1-6)-glucan in the fungal cell wall (Cabib et al., 2007). Biochemical studies on *P. brasiliensis* have demonstrated that chitin in the mycelium cell wall is exclusively connected through β-(1-3)-glucan (Kanetsuna et al., 1969).

We also found ECM homologues in *P. brasiliensis* with high expression in mycelium, such as ECM33, which is involved in key aspects of cellular wall morphogenesis and virulence in *C. albicans*. In *A. fumigatus*, the null mutant for ECM33 resulted in the rapid germination of conidia, an increase of cellular adhesion, and hyper virulence. Glucan elongation proteins, which are involved in the crossed connection of cellular wall components of fungi, are not only associated with the biosynthesis and morphogenesis for the correct incorporation of glucan molecules on the cellular wall, but are also necessary for virulence in *C. albicans* and *A. fumigatus* on a murine model of infection.

The analysis performed using recombinant proteins indicated that ORFs expressed in *S. cerevisiae* were reactive against sera from patients with PCM. Among these proteins, rPADG_02842 exhibited specific reactivity to PCM and showed no cross-reactivity with other fungal or unrelated diseases (**Figure 3**). However, the four ORFs evaluated in this study did not exhibit reactivity with patient serum PB7 (**Figures 3**, **4**, and data not shown). While ORFs rPADG_01494 and rPADG_06557 showed more activity for the patient with candidiasis (CA1) at around 50 kDa, ORFs rPADG_01494, rPADG_04617, and rPADG_06557 exhibited reactivity for histoplasmosis (H2) around 50 kDa (**Figure 4**, and data not shown). ORFs rPADG_04617 and rPADG_01494 showed reactivity in patients with aspergillosis (A1 and A2) (**Figure 4** and data not shown). All the recombinant proteins reacted with the serum of patients diagnosed with fungal pneumonia (C6). The presence of sugars in these proteins, similar to GPI-proteins from other fungi, may elicit an immune response and cause cross-reactivity with other fungal diseases. This observation explains the detection of fragments with a mass greater than that predicted for these proteins since these sugars can lead to the formation of higher molecular weight aggregates.

The protein products of ORFs PADG_02842, PADG_04617, and PADG_01494 have a reduced number of areas of predicted *O*-glycosylation, while ORF PADG_06557 has a higher number of this type of glycosylation, which can cause cross-reactivity and a greater number of bands than expected since the sugars present in proteins may lead to a stronger immune response. Studies conducted by Puccia & Travassos showed that carbohydrate epitopes present in the GP43 protein are responsible for over 85% of the reactions against sera from patients with PCM (Puccia and Travassos, 1991).

Previously, some research groups reported the use of *P. brasiliensis* antigens in the production of specific antibodies for disease diagnosis. For instance, Silveira-Gomes and colleagues (2011) showed that the latex agglutination test using an exoantigen pool from *P. brasiliensis* presents cross-reactivity to sera from patients with histoplasmosis (27%, 3/11), aspergillosis (27%, 4/15), and non-fungal diseases (22%, 11/49) (Silveira-Gomes et al., 2011). Taborda and Camargo (1994) showed that western blot analysis of the protein gp43 against sera of patients with PCM is effective in analyzing the reactivity of patient sera and can be performed in less time than immunodiffusion assays (Taborda and Camargo, 1994).

**Table 1** presents an overview of various studies conducted to develop immunodiagnostic tools for Paracoccidioidomycosis. Among these methods, three molecules have been well-characterized, namely GP43 (Puccia et al., 1986), PB27 (Fernandes et al., 2011), PB40 (Fernandes et al., 2012). Additionally, two potential heat shock proteins for diagnosing fungal infections are HSP30 and HSP60 (Cunha et al., 2002; de Bastos Ascenço Soares et al., 2008; Thomaz et al., 2014). The PCN (paracoccin) may also serve as a useful diagnostic tool for these fungi (Coltri et al., 2006).

**Table 1:** Protein characterization from *Paracoccidioides brasiliensis* can have the potential for developing immunodiagnostic tools.

Protein	Name	Reference
GP43	Glycoprotein 43	(Puccia et al., 1986)
PB27	Antigenic protein from *P. brasiliensis*	(Fernandes et al., 2011)
PB40	Calcineurin B homolog of *Neurospora crassa*	(Fernandes et al., 2012)
HSP30	Heat-Shock-Protein 30	(de Souza et al., 2021)
HSP60	Heat-Shock-Protein 60	(Izacc et al., 2001)
PCN	Paracoccin	(Coltri et al., 2006)
2842 (SOD3)	Superoxide dismutase	(Tamayo et al., 2016), this study
1494	Glycoside hydrolase (DCW1)	This study

All four recombinant proteins were fused to a GST-tag, which facilitates the purification of the recombinant protein. Furthermore, the study utilized western blot analysis to demonstrate that the recombinant proteins rPADG_02842 and rPADG_01494 exhibit specific reactivity with antibodies present in the serum of individuals with PCM. Notably, these proteins did not show any reactivity towards other fungal infections, as illustrated in **Figures 3**, **4**. The rPADG_02842 exhibited high specificity in recognizing sera from patients with PCM, while their reactivity to GST was low (**Figure 5E**). This study also observed cross-reactivity against sera from patients with histoplasmosis and aspergillosis. ELISA tests demonstrated that the four recombinant proteins exhibited cross-reactivity with the sera of patients with aspergillosis (**Figure 5**).

The presence of sugars in the analyzed proteins and other epitopes present in these proteins may explain the cross-reactivity against sera from patients infected with other fungal diseases. Taken together, our data suggest that the recombinant protein rPADG_02842 is a potential candidate for use in serological tests for the diagnosis of paracoccidioidomycosis.

## Conclusions

In conclusion, our study sheds light on the significant role of various proteins in fungal infections and their potential as both diagnostic markers and therapeutic targets for fungal diseases. Moving forward, further investigations should be directed towards understanding the precise mechanisms by which these proteins interact with the host immune system and contribute to the pathogenesis of fungal infections. Additionally, it is crucial to explore the expression of GPI-recombinant proteins in heterologous systems to thoroughly evaluate their diagnostic and immune potential, especially in distinguishing between different *Paracoccidioides* species. Ensuring specificity in diagnostic methodologies is essential for accurately identifying and managing fungal infections caused by different species within the *Paracoccidioides* genus.

## Data availability statement

The original contributions presented in the study are included in the article/supplementary material. Further inquiries can be directed to the corresponding author.

## Ethics statement

This study used serum samples obtained from patients as part of routine care. The Ethics Committee on Research at Hospital das Clínicas da FMRP-USP (protocol #13982/2005) did not require the study to undergo review or approval from an ethics committee because the study used sera from patients who had already undergone laboratory testing. The Hospital provided samples as part of a project, but the number of samples was limited, so the authorization obtained from the ethics committee was deemed sufficient.

## Author contributions

RG and IP contributed equally to this work. RG, IP, TR, LB, and PC contributed to conception and design of the study. RG, IP, TR, HZ, RM, and PC organized the database. RG, IP, HZ, and PC performed the statistical analysis. RG, IP, and PC wrote the first draft of the manuscript. RG, IP, TR, LB, MP, CS, RM, and PC wrote sections of the manuscript. All authors contributed to manuscript revision, read, and approved the submitted version.
